# Retraction Note: Identification of a novel microRNA-mRNA regulatory biomodule in human prostate cancer

**DOI:** 10.1038/s41419-026-08534-2

**Published:** 2026-03-06

**Authors:** Yanqiong Zhang, Funeng Jiang, Huichan He, Jianheng Ye, Xia Mao, Qiuyan Guo, Shu-lin Wu, Weide Zhong, Chin-Lee Wu, Na Lin

**Affiliations:** 1 Institute of Chinese Materia Medica, China Academy of Chinese Medical Sciences, Beijing, 100700 China; 2 Department of Urology & Pathology, Massachusetts General Hospital and Harvard Medical School, Boston, MA 02114 USA; 3 Department of Urology, Guangdong Key Laboratory of Clinical Molecular Medicine and Diagnostics, Guangzhou First People’s Hospital, Guangzhou Medical University, Guangzhou, 510180 China; 4 Urology Key Laboratory of Guangdong Province, The First Affiliated Hospital of Guangzhou Medical University, Guangzhou Medical University, Guangzhou, 510230 China

Retraction Note to: *Cell Death & Disease* 10.1038/s41419-018-0293-7; Article published online 21 February 2018

The Editors-in-Chief have retracted this article. After publication, concerns were raised regarding the data presented in the figures and supplementary materials, specifically:

- Fig. 3b (left) appears to overlap with Fig. 5g (left)
- A number of tumours in Fig. 3d and l appear to be highly similar with rotation and size changes
- Fig. 3g (right) appears to overlap with Fig. 5g (right)
- Fig. 4c (top right) appears highly similar to Fig. 6b (bottom right)
- Fig. 5h (bottom right) appears to overlap with Fig. 6c (bottom right)
- The western blots presented in Fig. S4b appear to have background inconsistencies
- Fig. S7b (left) appears to overlap with Fig. S8c (bottom right)
- Fig. S7f (left) appears highly similar to S9b (left) and S8c (bottom left)
- Fig. S7f (left) appears to have repeated patterns within the image
- Fig. S8c (top left) appears to have repeated patterns within the image
- Fig. S8d (bottom left) appears highly similar to Fig. S9c (bottom left) and S9g (bottom right)
- Fig. S10a western blots contain breaks in the backgrounds
- Fig. S10c appears to have repeated patterns within the image.

The Editors-in-Chief therefore no longer have confidence in the presented data.

Weide Zhong does not agree with this retraction. The other authors did not respond to any correspondence from the editor or publisher about this retraction.

